# The Royal Hospital for Incurables, Putney

**Published:** 1917-04-21

**Authors:** Helena President


*To the Editor of* The Hospital.

### The Royal Hospital for Incurables, Putney.

*To the Editor of* The Hospital.

Sir,—While the first claim on the generosity of the public belongs by right to those who have sacrificed so much for their country, it must never be forgotten that another large class of sufferers exists to whom no help comes by right, for whose heroism no V.C.s are given, and for whom the future seems unrelieved darkness illumed by no ray of glory or patriotism. These are the incurable cases for whom the Royal Hospital for Incurables, at Putney Heath, affords refuge and support. There will, it is feared, be a large increase in their numbers owing to the indirect effects of the war. Of these cases the most entirely unbefriended are helped by the Ladies' Association of the hospital, which, year after year, rescues from complete despair. I appeal earnestly on their behalf for £500 to carry on this urgently necessary work.

All contributions will be gratefully received by Miss Allcroft, the Honorary Secretary of the Ladies' Association, at 93 Elizabeth Street, Eaton Square, S.W. 1.

April 17, 1917. Helena, President.

